# A descriptive research: Exclusion from submitted clinical data package in the review process of new drug approval due to GCP violation in Japan

**DOI:** 10.1016/j.conctc.2019.100416

**Published:** 2019-07-19

**Authors:** Ryuta Asada, Kenichi Yoshimura, Kayo Hattori, Yujiro Nonaka, Hiroi Kasai, Shinobu Shimizu

**Affiliations:** a Innovative and Clinical Research Promotion Center, Gifu University Hospital, Gifu, Japan Clinical, Japan; b Innovative Clinical Research Center, Kanazawa University Hospital, Japan; c Institute for Advancement of Clinical and Translational Science (iACT), Kyoto University Hospital, Japan; d Department of Advanced Medicine, Nagoya University Hospital, Japan

**Keywords:** GCP, Clinical trial, GCP inspection, GCP violation

## Abstract

**Introduction:**

In Japan, the PMDA conducted inspections based on GCP in the review process of the submission of NDAs or sNDAs. In this descriptive study, we examined in detail the contents of exclusion data from submitted clinical data package subjects in the results of GCP inspections in Japan for NDAs or sNDAs.

**Methods:**

For NDAs or sNDAs approved in Japan between January 2007 and December 2017, we gathered information about the PMDA's conclusion from review reports, concerning the results of the GCP on-site inspection.

**Results:**

For 1193 NDAs and sNDAs approved in Japan between 2007 and 2017, there were 37 cases in 33 applications of non-compliance with GCP, including 1 by the sponsor and 32 at the clinical trial site. Of the 32 applications at the clinical trial site, 9 cases were categorized as “General findings” and 28 as “Findings for individual subjects.” Of the 9 “General findings” cases, problems related to the IRB were most common (44.4%), while faulty record keeping was the most common (60.7%, 95% confidence interval 42.6%–78.9%) problem in the 27 “Findings for individual subjects” cases. Violations of GCP were mostly found in 2007 and 2009, but few were found after 2013.

**Conclusion:**

In this study, we revealed that record keeping was the most common reason for exclusion from the analysis data of subjects in the results of GCP inspections. It is necessary to be careful in maintaining medical records, especially when conducting clinical trials without using electronic medical records.

## 1 Introduction

Clinical trials of human medicines for marketing authorization applications in Japan must comply with the ministerial ordinance on GCP. This requirement was based on the International Council for Harmonisation of Technical Requirements for Pharmaceuticals for Human Use (ICH) Guidelines E6 (Guideline for Good Clinical Practice) enacted in April 1998 [1].

In Japan, the Pharmaceuticals and Medical Devices Agency (PMDA) conducts GCP inspections during submission of new drug applications (NDAs) for marketing authorization or supplemental new drug applications (sNDAs) for extending the range of indication and/or posology, in addition to the method of administration. The PMDA is a Japanese regulatory agency sharing responsibilities with the Ministry of Health, Labour and Welfare (MHLW) of Japan. In contrast, in the European Union (EU), GCP inspections are usually requested during the initial review of a marketing authorization application (MAA), but could be requested post-authorization by the European Medicines Agency [2]. These inspections may be routine or may be triggered by issues arising during the validation of the pivotal clinical trials submitted to the EMA, through assessment of the application dossier by the assessors, or through other information such as previous inspection experience [2]. In the United States (US), GCP inspections are implemented by the US Food and Drug Administration (FDA) in the same manner as the EU. Watanabe et al. reported that the regulatory framework of GCP inspections in the US and EU make it possible for regulatory authorities to conduct on-going investigations of trials and also to conduct system-based investigations rather than submitted documents-based and accuracy-oriented investigations [3].

The conclusions of the GCP inspection in Japan were classified into three categories as “Compliance,” “Compliance with condition,” and “Non-compliance.” The first category means that the application dossier was acceptable, and if necessary, voluntary actions were indicated. The second means that the violation of GCP was confirmed for a portion of the subjects. Therefore, the application dossier can be accepted after excluding from the analysis the data in the NDA clinical data package for the NDAs or sNDAs. The third means that the GCP violation was found to be general and systematic. Therefore, the application dossier submitted is not reliable, and all clinical trial data should be deleted.

It is important to avoid deleting the data of subjects participating in clinical trials. To do so, it is important to understand why these data were recently eliminated from the NDAs or sNDA packages. To date, some studies have analyzed GCP deficiencies in submitted trials for NDAs or sNDAs approved by the MHLW of Japan from the 1990s to the 2000s [[4], [5], [6], [7], [8], [9], [10]]. A study has examined the quality in oncological clinical trials for NDA or sNDA in Japan, between April 2004 and March 2010 [11], and has indicated that the exclusion of patients from the review objective due to serious violations of GCP in 40 audits for oncology drug applications was observed in 2 (5.0%) cases, whereas that in the remaining 343 audits for drug applications of the other therapeutic area was observed in 40 (11.7%) cases. However, to our knowledge, there are no recent papers examining compliance with GCP in submitted trials for NDAs or sNDAs.

In this study, we examined in detail the contents of deleted subject data in the results of GCP on-site inspections in Japan for NDAs or sNDAs between January 2007 and December 2017. As a result, we aimed to reveal the considerations in clinical trial operations related to GCP when designing, conducting, recording, and reporting the trials.

## 2 Methods

We gathered review reports of NDAs or sNDAs approved in Japan between January 2007 and December 2017 from the official PMDA website. These reports contain information about PMDA's conclusion concerning the results of the GCP inspections. GCP on-site inspection in Japan is performed for the sponsor and clinical sites. For each NDA or sNDA, the following data were collected: the medicinal classification of the approved drug, approval year, and PMDA's judgment on GCP compliance.

For all, we extracted the NDAs or sNDAs for which PMDA classified into “Compliance with condition” at the conclusion of the inspections. Next, we examined the reason why PMDA made a judgment of non-compliance with GCP, and specified the responsible participants (sponsor or clinical trial site) due to deﬁciencies. We classified findings at the clinical trial site into “General findings” and “Findings for individual subjects,” and examined these in detail. We also examined the relationship between approval year and findings at the clinical trial site.

## 3 Results

From 2007 to 2017, 1193 NDAs or sNDAs were approved in Japan. GCP on-site inspections were conducted for 985 NDAs or sNDAs. However, inspections were not conducted for the other NDAs or sNDAs, because their data indicated that there were no clinical trials subjected to GCP on-site inspections. From the results of GCP on-site inspections in Japan, in the case of 33 NDAs or sNDAs, the subject data were removed from the submitted data due to GCP non-compliance. These results are shown in Table 1.

**Table 1:** Conclusion of GCP on-site inspections.

Year	Number of approved NDAs	Number of GCP on-site inspections	Number of NDAs with subjects' data deleted
2007	83	70	8
2008	79	65	4
2009	94	82	10
2010	109	91	3
2011	130	88	2
2012	121	88	2
2013	125	95	0
2014	132	114	1
2015	108	101	2
2016	125	110	0
2017	87	81	1
Total	1193	985	33

We examined the reason why PMDA judged the 33 NDAs or sNDAs as non-compliant with GCP. Of the 33, 1 was found to be non-compliant due to the sponsor and 32 were reported at the clinical trial site. In the former, the sponsor did not appropriately perform the duties related to preparation and management of the clinical trial, and may have caused a delay in providing safety information or made a false statement in the monitoring activities. On the other hand, we examined in detail, the latter 32 NDAs or sNDAs. The results revealed that findings in the clinical site could be classified into “General findings” and “Findings for individual subjects.” “General findings” were found in 6 NDAs or sNDAs and “Findings for individual subjects” in 25, while both were found in 1.

**Fig. 1:**
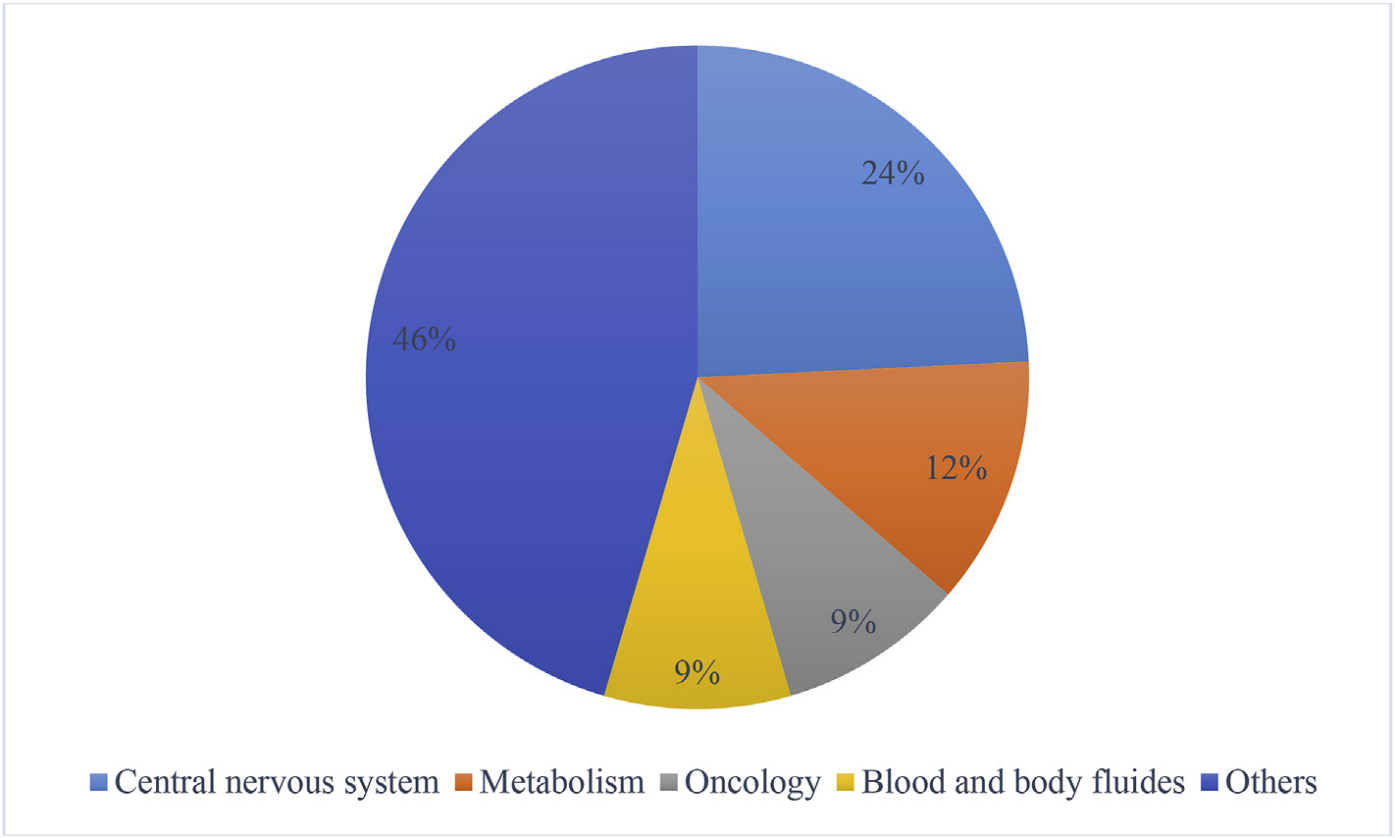
Therapeutic categories for the 33 NDAs or sNDAs.

The therapeutic categories for the 33 NDAs or sNDAs are shown in Fig. 1. Central nervous system was the most popular target, followed by metabolism, oncology, and blood and body fluids.

### 3.1 General findings

For 2 out of 7 NDAs or sNDAs, we found 2 findings each, and for the others, we found 1 each. Thus, for 9 findings, the details of “General findings” are shown in Table 2. Problems related to the Institutional Review Board (IRB) was the most common reason for non-compliance with GCP, followed by Investigator, and Control of Investigational Product. The details of problems related to “IRB” were that it did not maintain written records of its activities (2 NDAs), did not perform its functions according to written operating procedures, and did not been established according to the ministerial ordinance on GCP in Japan. The detail of problem related to “Investigator” was that he/she did not fulfil his/her responsibilities. The detail of problems related to “Control of Investigational Products” was that the investigator/institution did not file records of the use of investigational products by each subject.

**Table 2:** Details of general findings.

Reason	Number (Rate)
IRB	4 (44.4)
Investigator	3 (33.3)
Control of Investigational Products	2 (22.2)
Total	9 (100)

### 3.2 Findings for individual subjects

For 2 of 26 NDAs or sNDAs, we came up with 2 findings each, and for the rest, 1 each. Therefore, for 28 findings, the details of “Findings for individual subjects” are shown in Table 3. Record keeping was the most common reason for not including data, followed by selection of subjects, informed consent, and protocol deviations. Protocol deviations were defined as any content other than selection of subjects.

**Table 3:** Details of findings for individual subjects.

Reason	Number	Rate (95% confidence interval)
Record keeping	17	60.7 (40.6, 78.5)
Selection of subjects	6	21.4 (8.3, 41.0)
Informed consent	3	10.7 (2.3, 28.2)
Protocol deviations	2	7.1 (0.9, 23.5)
Total	28	100

The detailed contents of the 8 findings related to the selection of subjects (6 findings) and protocol deviations (2 findings) are shown in Table 4.

**Table 4:** Detailed Contents of Findings Related to Selection of Subjects and Protocol Deviations. The detailed contents of 3 findings related to informed consent are shown in Table 5.

Approval date	Non-proprietary name	Indication	Detailed contents
Oct. 2010	Polyethylene glycol treated human normal immunoglobulin	Improvement of muscle weakness in patients with chronic inflammatory demyelinating polyradiculoneuropathy (including multifocal motor neuropathy)	2 patients were proven not to comply with inclusion criteria (resistant to corticosteroids) owing to inconsistency between source documents and case report forms.
Oct. 2009	Aprepitant	Acute and delayed nausea and vomiting associated with cancer chemotherapy including cisplatin	One patient met exclusion criteria.
Apr. 2009	Sodium-potassium formulation Mosapride Citrate Hydrate	Bowel cleansing prior to barium enema X-ray examination	Principal investigator and 3 sub-investigators were registered as trial subjects.
July 2008	Mometasone furoate hydrate	Allergic rhinitis	For a subject, all hematologic tests were never implemented throughout the duration of clinical trial.
Oct. 2007	Gadoxetate Sodium	Contrast of liver tumors in magnetic resonance imaging (MRI)	Some patients did not meet inclusion criteria.
Oct. 2007	Nicorandil	Acute heart failure	One patient did not meet inclusion criteria (pulmonary artery wedge pressure >13 mmHg).
			For a subject, an investigational drug was administrated over the defined time period. For a subject, an excessive amount of an investigational drug was administrated.
Oct. 2007	Glucose and inorganic salts formulation	Irrigation and perfusion during surgery	2 patients participated in other clinical trials of 5-ALA at the same time.

**Table 5:** Detailed contents of findings related to informed consent and protocol deviations.

Approved in	Non-proprietary name	Indication	Detailed contents
June 2012	Axitinib	Advanced and metastatic renal cell carcinoma	Consent was not acquired again after supplying the subject with information that was relevant to the subject's willingness to continue participation in the trial.
Oct. 2009	Vancomycin	MRSA, MRSE	An impartial witness did not attend the informed consent process despite the subject having visual impairment.
Jan. 2009	Nalfurafine Hydrochloride	Improvement of pruritus in hemodialysis patients (for use only when conventional treatments are not sufficiently effective)	Consent had been obtained from a subject's legally acceptable representative but not authorized by the protocol and its details were also unknown.

### 3.3 Approval year and findings

For 36 findings at the clinical trial site, the relationship between approval year and findings is shown in Table 6. The GCP violations were mostly found in 2007 and 2009, and few violations were found after 2013.

**Table 6:** Approval year and 36 findings.

	2007	2008	2009	2010	2011	2012	2013	2014	2015	2016	2017
General findings	4	0	2	0	1	2	0	0	0	0	0
IRB	2	0	1	0	0	1	0	0	0	0	0
Investigator	1	0	1	0	0	1	0	0	0	0	0
Control of investigational products	1	0	0	0	1	0	0	0	0	0	0
Monitoring	0	0	0	0	0	0	0	0	0	0	0
**Finding for individual subjects**	**6**	**5**	**8**	**3**	**1**	**2**	**0**	**1**	**1**	**0**	**1**
Record keeping	2	4	4	2	1	1	0	1	1	0	1
Selection of subjects	3	0	2	1	0	0	0	0	0	0	0
Informed consent	0	0	2	0	0	1	0	0	0	0	0
Protocol deviations	1	1	0	0	0	0	0	0	0	0	0
**Total**	**10**	**5**	**10**	**3**	**2**	**4**	**0**	**1**	**1**	**0**	**1**

## 4 Discussion

For 33 (2.8%) out of 1193 NDAs or sNDAs approved in Japan between 2007 and 2017, there were 37 instances of GCP non-compliance findings; 1 by the sponsor and 32 at the clinical trial site. Of the 32 applications at the clinical trial site, 9 cases were categorized as “General findings” and 28 as “Findings for individual subjects.” In a previous study [9], there were 52 (19.8%) cases non-compliant with GCP out of 267 NDAs or sNDAs approved in Japan between April 1999 and March 2006. Therefore, though ICH-GCP were enacted in 1997, it was suggested that recent trials have become much better in quality than those conducted in the early 2000s.

Our descriptive study had certain limitations. We were not able to use the full on-site GCP inspection data, because the PMDA review reports are the only available data source and these do not contain detailed data. Cases in which pivotal trials in the NDA or sNDA package was conducted in foreign countries or multiple regions, the PMDA conducts on-site GCP inspection overseas [12]. However, it was impossible to distinguish whether GCP deficiencies occurred in Japan or foreign countries, because the institution that recognized GCP deficiencies was not clarified in the PMDA review reports. However, it was possible to evaluate considerations related to the implementation of clinical trials to avoid deleting the subject data.

We examined these 28 “Findings for individual subjects” in detail. Of 17 findings related to record keeping, 15 involved loss of medical records, and 2 involved loss of source documents related to the primary endpoint. In the case of the introduction of electronic medical records by the medical institution, there is a low possibility of loss of records. However, when conducting clinical trials at a medical institution that has not introduced electronic medical records, it is necessary to be careful about maintaining the records. It is important to research how to maintain medical records and include a record preservation period when selecting medical institutions for clinical trials.

Second, we examined in depth 8 findings related to the selection of subjects (6 findings) and protocol deviations (2 findings) (Table 4). Although it was not clearly indicated, the data suggest the need for carefully ensuring that a subject satisfies the inclusion criteria for the study, which is also related to the study's efficacy evaluation. It is also necessary to carefully check the accuracy and completeness of subject selection during monitoring and audit.

Third, we examined in detail, 3 findings related to informed consent (Table 6). The results suggested the need to be especially careful if the subject cannot read the relevant documentation supplied (for instance, the informed consent form), or if the consent of the subject's legally acceptable representative is required. It is important to check whether an impartial witness or the subject's legally acceptable representative needs to attend the informed consent process.

As shown in Table 6, the most GCP violations were found from 2007 to 2012, and few violations were found after 2013. As a reason for this tendency, there is a possibility that the effective structure of clinical trials was gradually ensured during the 2010s because “New 5-Year Clinical Trial Activation Plan” [13] was started by MHLW of Japan since April 2007.

## 5 Conclusion

The results of this study reveal that record keeping was the most common reason for deleting subject data from the submitted data in the results of GCP inspections. We revealed that it is necessary to be careful about maintaining medical records, especially when conducting clinical trials at medical institutions that have not introduced electronic medical records. We indicated the need for carefully ensuring that a subject satisfies the inclusion criteria for a study, which is also related to the study's efficacy evaluation. We also indicated the need to be especially careful if the subject cannot read the relevant documentation supplied (for instance, the consent form), or if the consent of the subject's legally acceptable representative is required.

The findings of GCP inspections may be useful for identifying risks when determining an appropriate risk-based monitoring plan. The clinical development of medicines is a global undertaking. Therefore, in the future, we would like to evaluate whether there are differences in the GCP inspection conclusions between the US, EU, and Japan.
